# RSF1 and Not Cyclin D1 Gene Amplification May Predict Lack of Benefit from Adjuvant Tamoxifen in High-Risk Pre-Menopausal Women in the MA.12 Randomized Clinical Trial

**DOI:** 10.1371/journal.pone.0081740

**Published:** 2013-12-19

**Authors:** Dana Keilty, Marguerite Buchanan, Katerina Ntapolias, Olga Aleynikova, Dongsheng Tu, Xiao Li, Lois Shepherd, Vivien Bramwell, Mark Basik

**Affiliations:** 1 Department of Oncology and Surgery, Lady Davis Institute, Jewish General Hospital, Montreal, Quebec, Canada; 2 Department of Pathology, Jewish General Hospital, Montreal, Quebec, Canada; 3 NCIC Clinical Trials Group, Cancer Research Institute, Queen's University, Ontario, Canada; 4 Department of Medical Oncology, Tom Baker Cancer Centre, Calgary, Alberta, Canada; University Hospital of Modena and Reggio Emilia, Italy

## Abstract

Most women with estrogen receptor expressing breast cancers receiving anti-estrogens such as tamoxifen may not need or benefit from them. Besides the estrogen receptor, there are no predictive biomarkers to help select breast cancer patients for tamoxifen treatment. CCND1 (cyclin D1) gene amplification is a putative candidate tamoxifen predictive biomarker. The RSF1 (remodeling and spacing factor 1) gene is frequently co-amplified with CCND1 on chromosome 11q. We validated the predictive value of these biomarkers in the MA.12 randomized study of adjuvant tamoxifen vs. placebo in high-risk premenopausal early breast cancer. Premenopausal women with node-positive/high-risk node-negative early breast cancer received standard adjuvant chemotherapy and then were randomized to tamoxifen (20 mg/day) or placebo for 5 yrs. Overall survival (OS) and relapse-free survival (RFS) were evaluated. Fluorescent in-situ hybridization was performed on a tissue microarray of 495 breast tumors (74% of patients) to measure CCND1 and RSF1 copy number. A multivariate Cox model to obtain hazard ratios (HR) adjusting for clinico-pathologic factors was used to assess the effect of these biomarkers on Os and RFS. 672 women were followed for a median of 8.4 years. We were able to measure the DNA copy number of CCND1 in 442 patients and RSF1 in 413 patients. CCND1 gene amplification was observed in 8.7% and RSF1 in 6.8% of these patients, preferentially in estrogen receptor-positive breast cancers. No statistically significant interaction with treatment was observed for either CCND1 or RSF1 amplification, although patients with high RSF1 copy number did not show benefit from adjuvant tamoxifen (HR = 1.11, interaction p = 0.09). Unlike CCND1 amplification, RSF1 amplification may predict for outcome in high-risk premenopausal breast cancer patients treated with adjuvant tamoxifen.

## Introduction

About three quarters of breast cancers express the estrogen receptor (ER), which renders them susceptible to treatment by modulators/antagonists of ER activity such as the ER antagonist, tamoxifen. Presently, treatment of ER-expressing (ER+) breast cancers by anti-estrogen drugs is the standard of care, with survival benefit even in very early breast cancers, as shown by the NSABP B-14 clinical trial in node-negative ER+ patients [1]. Since all women with high-risk tumors (e.g. presenting with axillary lymphatic metastases, or high grade or large tumor size) receive chemotherapy in the adjuvant setting, there was a question regarding the added benefit of tamoxifen to that of chemotherapy. The MA.12 clinical trial was designed to answer the question of whether tamoxifen adjuvant therapy was also of benefit in pre-menopausal women with early (pathologic stage I-III) but high-risk (high-risk node-negative or node-positive) breast cancer after the administration of cytotoxic chemotherapy. The results of this trial were recently reported: it was found that tamoxifen improved disease-free survival in these higher-risk early breast cancer patients, albeit not statistically significantly [2]. This apparent lack of significant benefit may in part be due to the poor overall added activity of tamoxifen over and above that of adjuvant chemotherapy. However, it is also possible that a poor selection of potential candidates of tamoxifen therapy may have contributed to these counter-intuitive results, in that the benefit obtained from tamoxifen therapy in a subset of patients may have been obscured by the lack of benefit in another subset. The search for candidate biomarkers of tamoxifen response will help to better define subsets of early breast cancer patients in whom tamoxifen therapy may either be withheld or administered with a greater certainty of its relative ineffectiveness or benefit.

The genomic analysis of ER+ breast tumors confirms their lower clinical aggressiveness. Indeed, ER+ tumors show fewer and less complex genomic alterations, including DNA copy number gains (amplifications) or losses (deletions) [3]. However, some characteristic genomic alterations appear to be specific to these tumors. A frequently-observed DNA amplification is found on chromosome 11q13, which involves the cyclin D1 gene [4]. Cyclin D1 is a member of the cyclin family of cell cycle regulators, and its overexpression favors more rapid and sustained proliferation of breast tumor cells. The CCND1 gene, which encodes for cyclin D1, is amplified in approximately 10-15% of breast cancers, most of which are ER+ [4]. Cyclin D1 has been shown to be a factor in resistance to tamoxifen in pre-clinical studies [5]. However, this biomarker of resistance has not been validated in large randomized clinical trials of tamoxifen, and so is not in clinical use. Interestingly, the 11q13 amplicon is not the only amplified region on the long arm of chromosome 11: the 11q14 region is also amplified, albeit less frequently than 11q13. Moreover, this region is frequently co-amplified in tumors that contain the 11q13 amplicon. The target of this second amplicon has been thought to be the PAK1 gene, which encodes a p21-driven kinase [6]. Our analysis of a tumor containing an 11q14 amplicon showed that PAK1 was not part of this amplicon, but that another interesting gene, RSF1, was contained within it. RSF1 is amplified and overexpressed in 25% of high-grade ovarian serous carcinomas [7] and codes for remodeling and spacing factor 1 (Rsf-1), a chromatin remodeling protein involved in the regulation of gene expression and cellular proliferation. It is associated with poor outcome in ovarian carcinoma [8] but has not been studied in breast cancer. We assessed the role of CCND1 and RSF1 gene amplification as candidate biomarkers predicting the benefit of adjuvant tamoxifen in the MA.12 clinical trial. Our results suggest that amplification of RSF1 – but not that of the gene encoding for cyclin D1 – may be a candidate predictive biomarker for adjuvant tamoxifen benefit in this high-risk early breast cancer population. 

## Methods

### Ethics Statement

Participants provided written informed consent to participate in the MA.12 study. The consent for correlative sciences work on the MA.12 samples was not part of the original consent and samples were collected in the late 1990's before explicit consent was required. Tissue blocks were requested from all participating Canadian hospital pathology departments after patient enrollment in the study. Ethics committee approval for this study on the MA.12 samples (including any and all samples from the 1990s) was obtained from the ethics committee of the Jewish General Hospital.

### Materials

The MA.12 clinical trial was a phase III randomized controlled clinical trial of adjuvant tamoxifen in pre-menopausal women with high-risk, early breast cancer. The MA.12 tissue microarray was created at Queen’s University from samples collected from pathology departments in hospital centers participating in the trial. The tissue collection had not been made mandatory to the trial. 

There were no significant differences between this subgroup and the entire study group in terms of patient characteristics (results not shown).

### Methods

#### Fluorescent in-situ hybridization (FISH)

FISH was performed to visualize CCND1 and RSF1 gene statuses. Bacterial artificial chromosome (BAC) clones specific to the two genes of interest were ordered from BACPAC Resources Centre (Oakland, CA) (CCND1 RP11-300I6, RSF1 RP11-1081L7 and RP11-1107J12). Two clones were ordered for RSF1 as per Brown et al. [8], in which both probes were required to visualize a signal bright enough to read. In our hands, the use of both probes was deemed redundant on the MA.12 TMAs and only the RP11-1107J12 probe was used.

Purified DNA from BACs was nick translated using a commercially-available nick translation kit (Abbott Molecular, Mississauga, ON) to directly label DNA with a fluorochrome conjugated to dUTP (Bayani & Squire, 2001). Orange dUTP (Inter Medico, Markham, ON) was used for both CCND1 and RSF1. Human COT-1 DNA (Invitrogen, Burlington, ON) and salmon sperm DNA (Invitrogen, Burlington, ON) were then added and the DNA precipitated in 100% ethanol and 3M sodium acetate. The mixture was first frozen at -80°C for at least 20 minutes and then centrifuged at 4°C for 10 minutes at 10 000 RPM. The supernatant was removed and the DNA pellet was air-dried in the dark. It was then resuspended with a SpectrumGreen chromosome-11 centromere enumeration probe (CEP11) (Abbott Molecular, Mississauga, ON) as per manufacturer’s instructions. Immediately before application to the slide during the FISH protocol, the probes were denatured at 73°C for 5 minutes.

TMA slides were first deparaffinised, then 0.2N hydrochloric acid was used to permeabilize the tissues for 30 minutes. They were then pre-treated in citric acid pre-treatment solution (Zytovision, Bremerhaven, Germany) for 1.5 hours. 0.5g of pepsin in 0.2N HCl was used as a protease solution, and proteolysis was performed at 37°C for 90 minutes, in new solution every 15 minutes. DAPI II counterstaining (Abbott Molecular, Mississauga, ON) was used to ensure this digestion was sufficient. The slide was stabilized in 4% paraformaldehyde in PBS and then denatured in formamide denaturation buffer at 73°C for 6 minutes. After dehydration in a 75%-90%-100% ethanol series, the probe mixture was hybridized in a dark, humidified hybridizing chamber at 37°C for 18 hours. 

The slides were then washed in an IGEPAL (Sigma-Aldrich, Oakville, ON) wash buffer in the dark, excess probe removed in super pure formamide (Fisher Scientific, Ottawa, ON) wash solution heated to 43°C for 10 minutes X3. The slides were then dried in the dark and counterstained with DAPI II. 

#### FISH Scoring

The Metafer (MetaSytems Group Inc., Waltham, MA) slide scanning system was used to automatically count the average of the ratios of orange to green signals as per manufacturer’s instructions. TMA cores were counted manually (visually) when the Metafer scanner failed (too few tiles counted, or DAPI stain too weak), using a Jenco Epi-fluorescent Microscope (Model No. EPI-F223, Jenco International Inc., Portland, OR) in the Department of Pathology, Jewish General Hospital. At least two areas from each core were used to count the orange to green ratios of 15 nuclei. The ratios of the 15 nuclei were averaged for each core. This number was considered the amplification state score for the core. 

#### Array CGH

Array CGH was performed on DNA extracted from a breast tumor bank at the Jewish General Hospital. Invasive breast carcinoma specimens were surgically resected from patients at the Jewish General Hospital (Montreal, Canada). Informed consent was obtained to collect breast tumor samples for research purposes. The banking of breast tumor surgical samples was approved by the Ethics Committee of the Jewish General Hospital. Array CGH was carried out as per our previously published work [9] using 1 ug of tissue DNA.

#### Data analysis

Relapse-free survival (RFS) and overall survival (OS) in MA.12 was defined respectively as the, time from randomization to earliest date of recurrence or death or censored on the last date the patient was known to be alive, and time from randomization to date of death or censored on the last date the patient was known to be alive. The baseline characteristics of patients with amplified and non-amplified CCND1 or RSF1 were compared using a chi-square test if they were categorical and Wilcoxon test if continuous. For the assessment of the prognostic value of CCND1 or RSF1 amplification, both RFS and OS of patients with amplified and non-amplified CCND1 or RSF1 were described by Kaplan-Meier curves and compared by the a multivariate Cox model adjusting for treatment, age, Eastern Cooperative Oncology Group (ECOG) performance status, time from diagnosis to randomization, nodal status, t-stage, receptor status, type of chemotherapy treatment. A multivariate Cox model including an additional interaction term between treatment and CCND1 or RSF1 amplification status was used to assess for predictive values of CCND1 or RSF1 amplification in RFS and OS. All statistical tests were two sided. 

## Results

### CCND1 and RSF1 amplification

**Figure 1:**
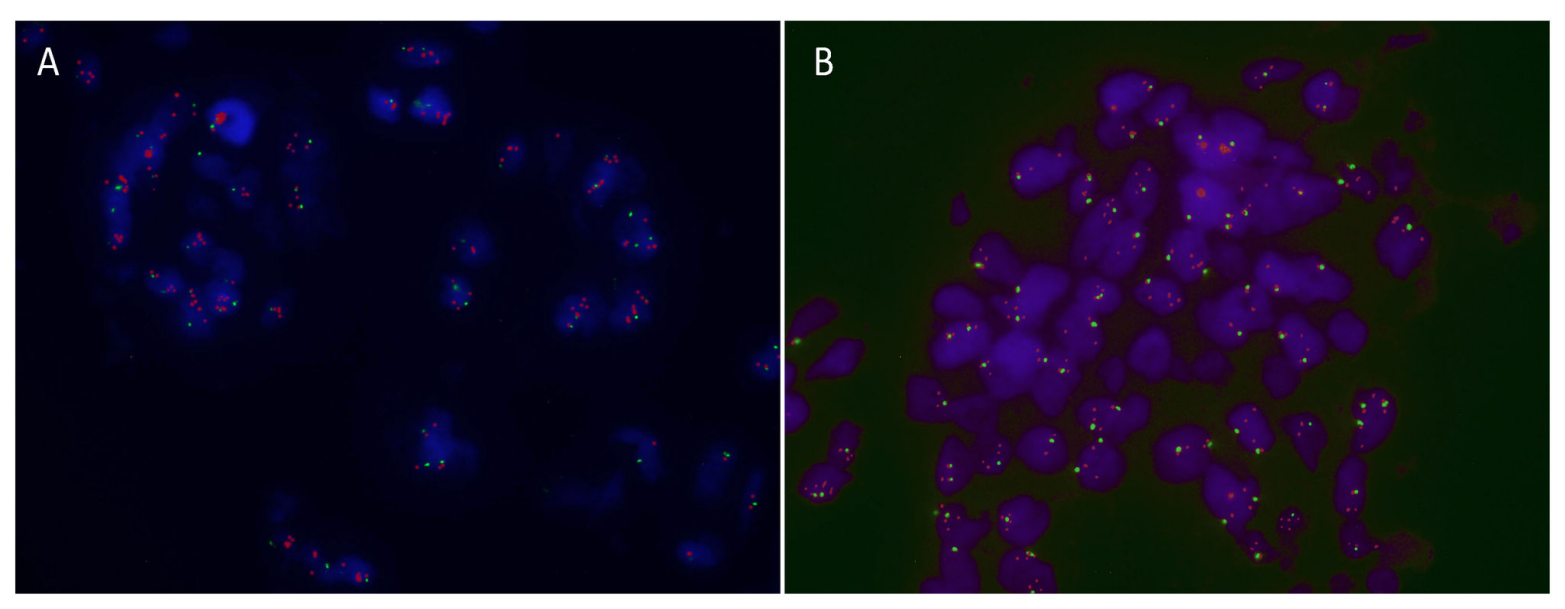
CCND1 and RSF-1 gene amplification by FISH. Images (100X magnification) of FISH for CCND1 (A) and RSF-1 (B) on two different breast tumor samples from the MA.12 tissue microarray. Sample A shows CCND1 gene amplification while sample B shows RSF-1 amplification. Green dots are centromeric probes for chromosome 11, while red dots represent the gene probes. Counterstaining is with DAPI II.

A total of 495 patients from the MA.12 clinical trial had tumors with representative single cores on the MA.12 TMA. We performed FISH using a BAC probe for the CCND1 gene and a corresponding commercially-available chromosome 11 centromeric probe. We validated the methodology on a breast tumor for which array comparative genomic hybridization (CGH) had shown focal amplification of CCND1 (Figure 1, Figure S1). 442 tumors had detectable probe values using the Metafer automated scoring system. We found that 38 (8.7%) tumors had FISH ratios ≥ 1.8, a cut-off used clinically for equivocal FISH scores in the case of ERBB2 (HER2) gene amplification [10]. Of these 38 tumors, 26 had FISH scores > 2.2, the clinically acceptable value for unequivocal gene amplification in the case of ERBB2 gene assessment. These values are similar to, albeit slightly lower than, those previously reported. 

**Figure 2:**
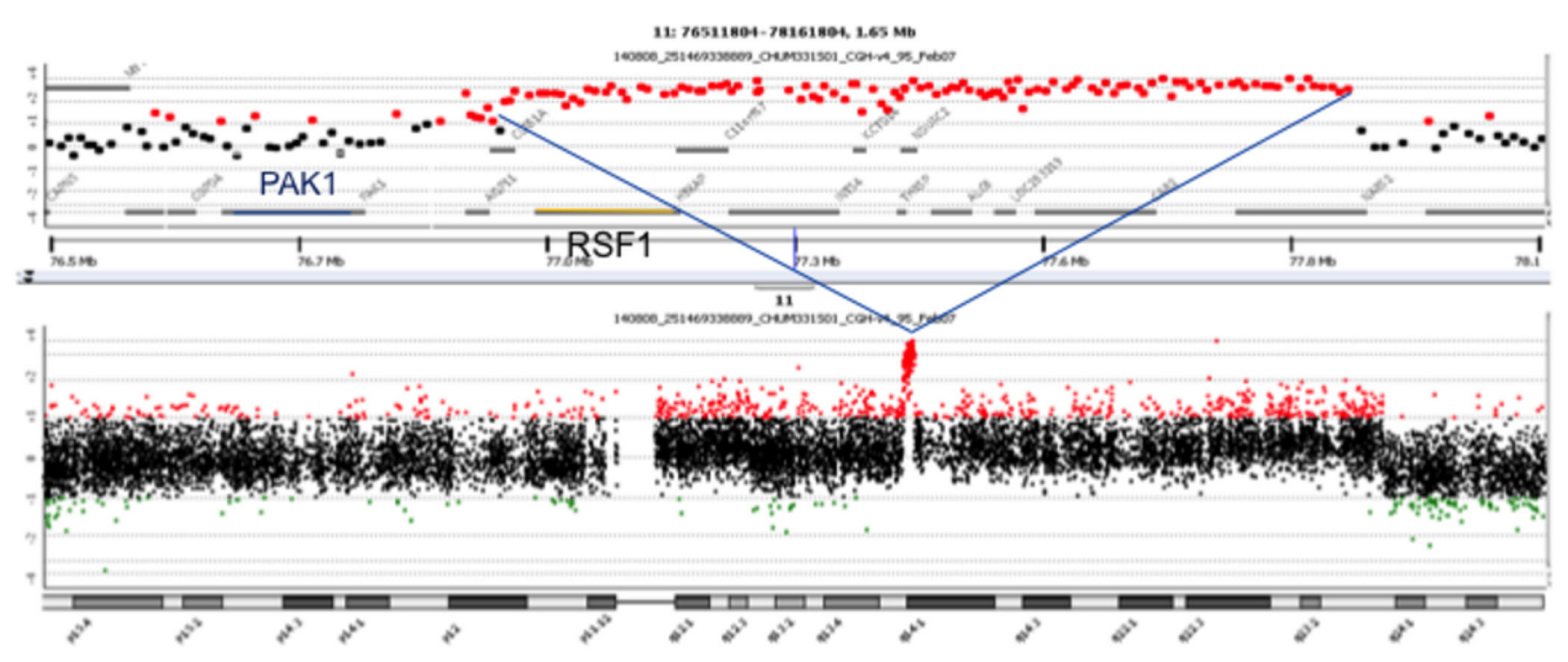
RSF-1 amplification by array CGH. Array CGH of tumor with chromosome 11q14 amplification that includes RSF1 and not PAK1. Top: focused view of 1.65 MB segment of chromosome 11q14 with genomic location of RSF1 (yellow bar) and PAK1 (blue bar) indicated. Red dots correspond to probes showing increased DNA copy number above log_2_=1. Green dots correspond to probes showing decreased DNA copy number below log_2_=-1. Bottom: a chromosome 11 view of the same amplicon from the same tumor.

Since it has been shown that 11q13 amplification is frequently associated with contiguous or distinct amplification of the neighboring 11q14 region, and that this region contains a candidate tamoxifen-resistance gene, p21-activated kinase-1 (PAK1) [6], we verified the presence of 11q14 amplification in an in-house array CGH dataset of 90 breast tumors. Nine of these tumors showed 11q14 amplification, most of them co-amplified with CCND1, as expected. However, one of these tumors in which CCND1 (11q13) was not amplified showed a very narrow 11q14 amplification, which on closer inspection, did not contain the PAK1 gene (Figure 2). It did contain the RSF1 gene, whose overexpression and amplification in ovarian cancer is a marker of poor prognosis. We decided to focus our attention on RSF1 amplification. FISH probes were created, and we measured RSF1 amplification status in 413 tumors from the MA.12 TMA (Figure 1). We found that 28 (6.8%) of all tumors showed RSF1 gene FISH scores ≥ 1.8, while 17 (4%) showed FISH scores > 2.2. 

Using the 1.8 score cut-off to define amplification, we found that 17 of the 27 RSF1 amplified tumors for which CCND1 amplification status was available showed co-amplification of CCND1. Thus, as expected, RSF1 and CCND1 genes were frequently co-amplified in this cohort of breast tumors. 

Because of the limited numbers of amplified patients and the continuous nature of the automated FISH score readings, we performed the outcome analysis using 1.8 as a cut-off for amplification for both CCND1 and RSF1 genes.

### Prognostic and predictive value of CCND1 amplification

As mentioned above, 400 (91.3%) patients were classified into the non-amplified CCND1 group and 38 (8.7%) into the amplified CCND1 group. Table S1 presents baseline characteristics for patients with respectively amplified and non-amplified CCND1. As expected, the CCND1-amplified subset had higher rates of ER positivity (84% vs. 63% for the non-amplified group, p=0.01). There was also a small difference in ECOG performance status, in that more ECOG-0 patients were in the non-amplified subgroup (p=0.04). 

**Figure 3:**
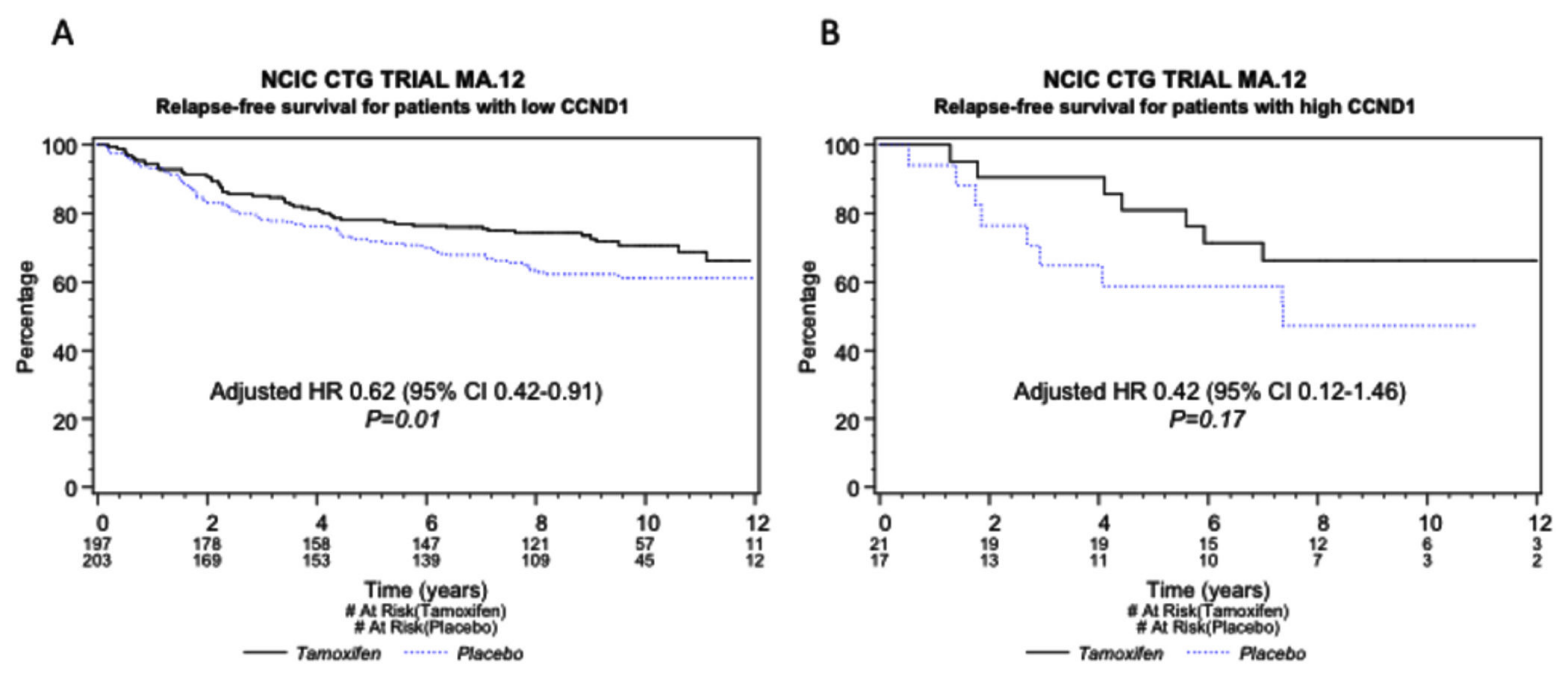
CCND1 amplification and relapse-free survival. RFS for patients with CCND1 gene amplification or not, showing benefit from tamoxifen in both groups. Kaplan-Meier curves for RFS, tamoxifen vs. placebo in patients with CCDN1 gene non-amplified (“low CCND1”) (A) and amplified (“high CCND1”) (B) tumors. Adjusted HRs are included for the comparison of tamoxifen vs. placebo in each group.

As expected, the presence of CCND1 amplification was associated with a trend toward poorer RFS in the entire cohort (HR 1.47, 95% CI 0.85–2.57, p=0.17), although this was not significant. The presence of CCND1 amplification was not associated with an OS benefit of tamoxifen over placebo (data not shown). In patients without CCND1 amplification, however, we found a significant relapse-free benefit of tamoxifen over placebo (HR 0.62, 95% CI 0.42–0.91, p=0.01), which was similar to that observed in patients with CCND1 amplified tumors (HR 0.42, 95% CI 0.12–1.46, p=0.17), though not significant. The p-value for interaction was 0.90 (Table 1). The Kaplan-Meier curves for RFS by tamoxifen treatment are shown in Figure 3 for patients with non-amplified (A) and amplified (B) CCND1. These findings suggest that, although CCND1 gene amplification may be a potential prognostic biomarker for RFS, it may not be a good predictive biomarker for adjuvant tamoxifen benefit or lack thereof in these high-risk pre-menopausal women, and should not be used as a predictive biomarker for tamoxifen in this group of breast cancer patients until other large clinical trial cohorts are examined. 

**Table 1:** RFS by treatment arm and CCND1 status.

CCND1 status and treatment		# of patients	5-year RFS (95% CI)	Adjusted Hazard Ratio (95% CI) [p-value]	P-value for interaction
Low CCND1	Tamoxifen	197	0.78 (0.72, 0.83)	0.62 (0.42, 0.91) [0.01]	0.90
	Placebo	203	0.72 (0.65, 0.77)		
High CCND1	Tamoxifen	21	0.81 (0.57, 0.92)	0.42 (0.12, 1.46) [0.17]	
	Placebo	17	0.59 (0.33, 0.78)		

“Low CCND1” status refers to patients with no CCND1 amplification; “High CCND1” status refers to patients with CCND1 amplification.

### Prognostic and predictive value of RSF1 amplification

As mentioned above, 381 (93.2%) patients were classified into the RSF1 non-amplified group and 28 (6.8%) into the RSF1-amplified group. Table S2 presents baseline characteristics for patients with respect to RSF1 gene amplification status. As was seen for CCND1, the RSF1-amplified subset had higher rates of ER positivity (82% vs. 64% for the non-amplified group, p=0.05). There were no other differences in clinico-pathological characteristics between the two groups. 

**Figure 4:**
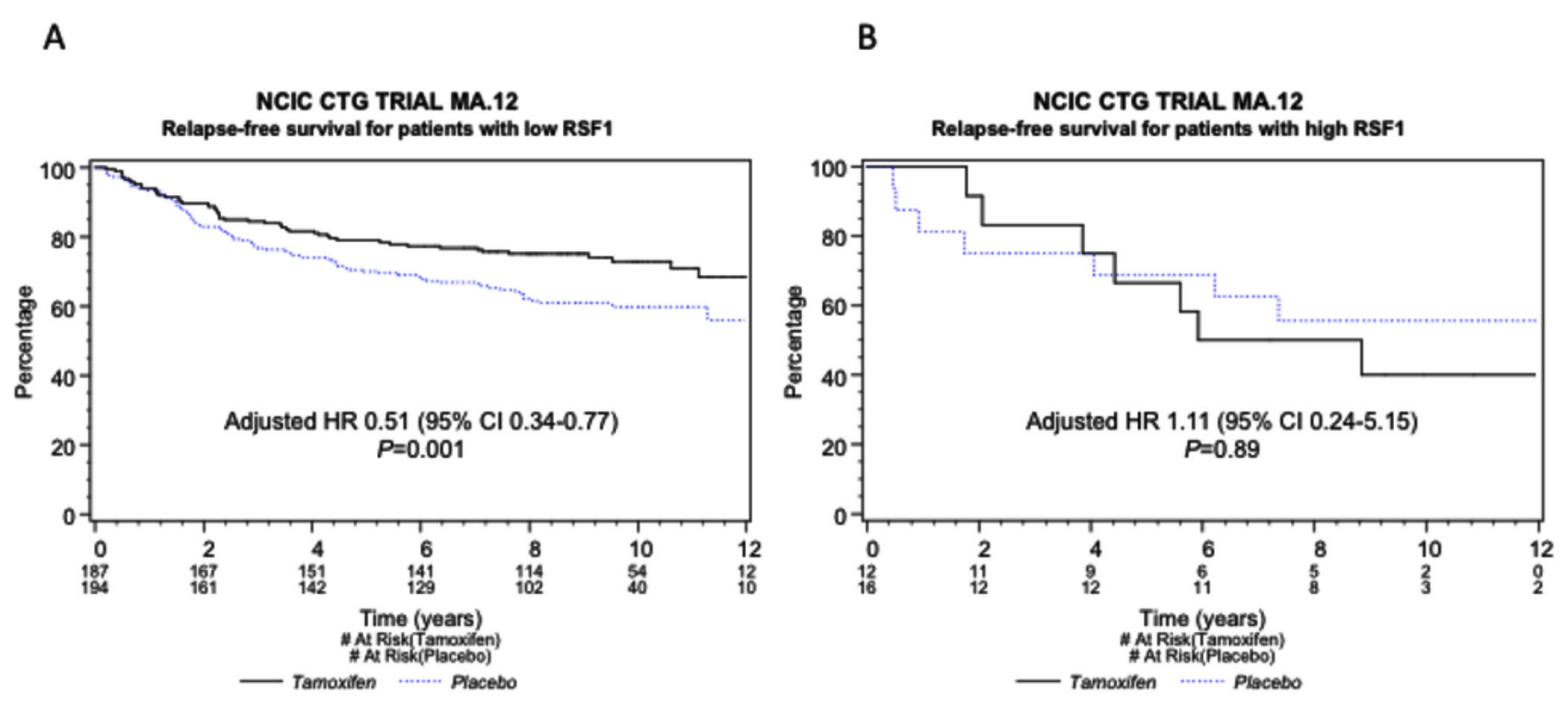
RSF1 amplification and relapse-free survival. RFS for patients with RSF1 gene amplification or not, showing benefit from tamoxifen in both groups. Kaplan-Meier curves for RFS, tamoxifen vs. placebo in patients with RSF1 gene non-amplified (“low RSF1”) (A) and amplified (“high RSF1”) (B) tumors. Adjusted HRs are included for the comparison of tamoxifen vs. placebo in each group.

There were non-significant trends toward poorer OS (HR 1.46, 95% CI 0.82–2.68, p=0.29) and RFS (HR 1.48, 95% CI 0.72–2.98, p=0.19) in the RSF1 amplified vs. the non-amplified group. The presence or lack of RSF1 amplification was not associated with an OS difference when tamoxifen was compared to placebo (data not shown). A lack of RSF1 amplification was, however, associated with a marked RFS benefit of tamoxifen over placebo (HR 0.51, 95% CI 0.34–0.77, p=0.001), which was in contrast to the lack of RFS benefit of tamoxifen over placebo in the RSF1-amplified group (HR 1.11, 95% CI 0.24–5.15, p=0.89). The p-value for interaction was 0.09 (Table 2). Kaplan-Meyer curves are shown in Figure 4. Although not statistically significant, this trend is suggestive of a role for RSF1 gene amplification as a novel candidate predictive biomarker for lack of benefit from adjuvant tamoxifen in these high-risk pre-menopausal women. 

**Table 2:** RFS by treatment arm and RSF1 status.

RSF1 status and treatment		# of patients	5-year RFS (95% CI)	Adjusted Hazard Ratio (95% CI) [p-value]	P-value for interaction
Low RSF1	Tamoxifen	187	0.79 (0.72, 0.84)	0.51 (0.34, 0.77) [0.001]	0.09
	Placebo	194	0.70 (0.63, 0.76)		
High RSF1	Tamoxifen	12	0.67 (0.34, 0.86)	1.11 (0.24, 5.15) [0.89]	
	Placebo	16	0.69 (0.40, 0.86)		

“Low RSF1” status refers to patients with no RSF1 amplification; “High RSF1” status refers to patients with RSF1 amplification.

Finally, we examined the predictive value of either RSF1 or CCND1 amplification: the p-value for interaction was 0.53 for both OS and RFS. We also examined the predictive value for tumors with both RSF1 and CCND1 amplification: in this case, the p-value for interaction was 0.23 for OS and 0.28 for RFS. 

## Discussion

Despite extensive use of anti-estrogen drugs like tamoxifen, there is, as of yet, no biomarker to assist the clinician in selecting patients who have a high probability of benefit from tamoxifen besides the measurement of ER and PR expression in the tumor tissue. Although the addition of tamoxifen markedly improved recurrence-free survival at 15 years follow-up from 65% to 78% in the NSABP B-14 study of women with ER+ early breast cancer, many of these patients did not benefit from tamoxifen [10]. The majority (65%) of these patients did not show disease recurrence when taking placebo, while 22% of patients eventually recurred despite tamoxifen therapy. There is clearly a need for a better biomarker for tamoxifen in the adjuvant setting in breast cancer. 

Several small studies have found candidate biomarkers predictive of response to tamoxifen, but none of these have been validated in large-scale prospective clinical trials. Among the most prominent of these are CYP2D6 polymorphisms and CCND1 (cyclin D1) amplification. 

The CYP2D6 story is particularly informative, exemplifying both the difficulty and the necessity of biomarker validation in large-scale prospective clinical trials. Although early and exciting evidence from small clinical trials strongly suggested that CYP2D6 genotyping predicted response to tamoxifen [11], these findings were not confirmed when CYP2D6 polymorphisms were assessed in two large randomized clinical trials (BIG-I-98 and ATAC) [12,13]. 

CCND1 amplification on chromosome 11q13 occurs in about 10% of breast cancer, and the associated cyclin D1 overexpression [14] has been correlated with a lack of response to tamoxifen [4]. Moreover, a neighboring amplicon in the chromosome 11q14 region, which is frequently co-amplified with the 11q13 region, appears to have even better predictive value for tamoxifen treatment, demonstrated in a small randomized trial in which patients were treated with only 2 years of tamoxifen [5]. Based on these results, Bostner et al. [5] suggested that the PAK1 gene is the target of that amplicon in breast cancers. We set out to bring this work to a more advanced level of validation by testing the predictive value of both amplicons in samples from a randomized clinical trial. 

One of the problems encountered when pursuing the validation of candidate predictive biomarkers was the dearth of clinical trials in which tamoxifen was compared to placebo, which is the optimal clinical trial design as it enables the distinction between predictive and prognostic value. Although a recent report showed that cyclin D1 gene amplification and expression have prognostic but not predictive value in material from a large clinical trial (Trans-ATAC) [15], which compared the aromatase inhibitor anastrozole to tamoxifen in the adjuvant setting, the absence of a placebo arm in that trial prevents any statement about the predictive value of cyclin D1 amplification or overexpression for anti-estrogen therapy. The one conclusion that can be drawn from that report is that, if CCND1 amplification did have negative predictive value, it would likely be equivalent in patients taking either anastrozole or tamoxifen. The National Cancer Institute of Canada Clinical Trials Group (NCIC-CTG) MA.12 clinical trial was placebo-controlled, and designed to study the value of administering tamoxifen in pre-menopausal women who had already received adjuvant chemotherapy in the context of high-risk early breast cancer. 672 patients were randomized to tamoxifen or placebo, and, with a follow-up of 9.7 years, there was no significant OS benefit from the administration of tamoxifen (HR 0.78, p=0.12) and a marginal benefit in disease-free survival (HR 0.77, p=0.056). 

Before testing the 11q13 and 11q14 amplicons as predictive biomarkers, we verified the extent and size of these amplicons in our in-house array CGH dataset of 90 breast tumors. In one breast tumor, we found a very narrow 11q14 amplicon that did not include the PAK1 gene. Of the ten genes contained in this 11q14 amplicon, the RSF1 gene was of great interest, as its over-expression and amplification had been implicated as a poor prognostic marker in ovarian cancers [7]. Shih et al. [16] found amplification at 11q13-14 in three of seven ovarian carcinomas, and RSF1 was the only gene the protein of which was consistently overexpressed in all tumors harboring the amplification. Patients with RSF1 amplification or overexpression had significantly shorter OS than those without. 

In the present study, CCND1 gene amplification did not appear to be confirmed as a predictive biomarker for adjuvant tamoxifen in high-risk breast tumors in pre-menopausal patients in the MA.12 study. Only 38 tumors showed CCND1 amplification in the MA.12 cohort, and this relatively small number somewhat limits the strength of the finding. Nevertheless, as this was the first time that CCND1 gene amplification was assessed in a large randomized clinical trial in the adjuvant setting, and the p-value for interaction is 0.90, our negative finding is noteworthy. This result was somewhat surprising, given previous reports suggesting that CCND1 amplification is one of the cardinal features of aggressive ER+ breast cancers, that the expression of the CCND1 gene is induced by estrogens, and that its amplification affects clinical response to tamoxifen. It is possible that the fact that the MA.12 patients had all received chemotherapy first may have blunted the role of cyclin D1 in regulating response to tamoxifen. Indeed, the higher proliferative state induced by increased levels of cyclin D1 expression may have led to augmented chemotherapy efficacy in these tumors, thereby neutralizing the previously-reported resistance of these tumors to tamoxifen. 

On the other hand, the amplification of the RSF1 gene emerged as a novel candidate biomarker that may be predictive of the lack of tamoxifen benefit in this clinical context. The significance of the interaction (p=0.09) was perhaps limited by the small number of samples that showed RSF1 amplification, even with the liberal threshold of a FISH ratio of 1.8. Nevertheless, it is telling that the hazard ratio (HR) for non-RSF1-amplified tumors was 0.51 with a p-value of 0.001. Although the p-value for interaction was not significant, we feel that this result warrants further high-level confirmation. 

Rsf-1 (remodeling and spacing factor 1) is a subunit of a chromatin assembly factor, RSF. It acts as the histone chaperone, combining with SNF2H, which has nucleosome-dependent ATPase activity [17]. The RSF chromatin assembly factor was initially identified in human cells as a “remodeling” factor that allows the formation of competent transcription initiation complexes on chromatin templates [18]. The RSF chromatin assembly factor thus plays an important role in regulating gene transcription. At present, there is no published data linking the function of Rsf-1 with ER activity. However, ample data suggest that chromatin remodeling activity regulates the activity of hormone receptors, and ER in particular [19]. At high levels of expression, such as that enabled by gene amplification, RSF1 may interfere with tamoxifen response. Several studies have linked this factor with various cancers, but none with breast cancer in particular. 

## Conclusion

Based on our findings, further study of the role of RSF1 in breast cancer biology, and, specifically, response to anti-estrogen therapies, is warranted. Further investigations into the negative predictive value of 11q14 in placebo-controlled studies, such as the NSABP B-14, are required to confirm the role of RSF1 amplification as a biomarker of negative predictive value for tamoxifen benefit in the adjuvant setting in early breast cancer. 

## Supporting Information

Figure S1 **CCND1 amplification validation.** Validation of a tumor with CCND1 gene amplification by FISH using array CGH data from DNA of the same tumor. FISH performed on a section from a paraffin-embedded formalin-fixed sample of a breast tumor (A) with red dots representing the CCND1 gene probe and green dots representing the centromeric 11q probe. (B) shows a chromosomal segment of chromosome 11 from a breast tumor that showed focal amplification of 11q13 including the CCND1 gene by array CGH (B). Red dots indicate probes with DNA copy number gain (values > log_2_=1).
(TIF)

Table S1 **Patient characteristics by CCND1 status.** **B**aseline characteristics for patients with non-amplified (“Low CCND1”) and amplified (“High CCND1”) CCND1.
(PDF)

Table S2 **Patient characteristics by RSF1 status.** Baseline characteristics for patients with non-amplified (“Low RSF1”) and amplified (“High RSF1”) RSF1.
(PDF)
