# Supplementary figures and images for: RSF1 and Not Cyclin D1 Gene Amplification May Predict Lack of Benefit from Adjuvant Tamoxifen in High-Risk Pre-Menopausal Women in the MA.12 Randomized Clinical Trial
Source: PLoS One. 2013 Dec 19;8(12):e81740. doi: 10.1371/journal.pone.0081740 (PMC3868649; doi:10.1371/journal.pone.0081740)

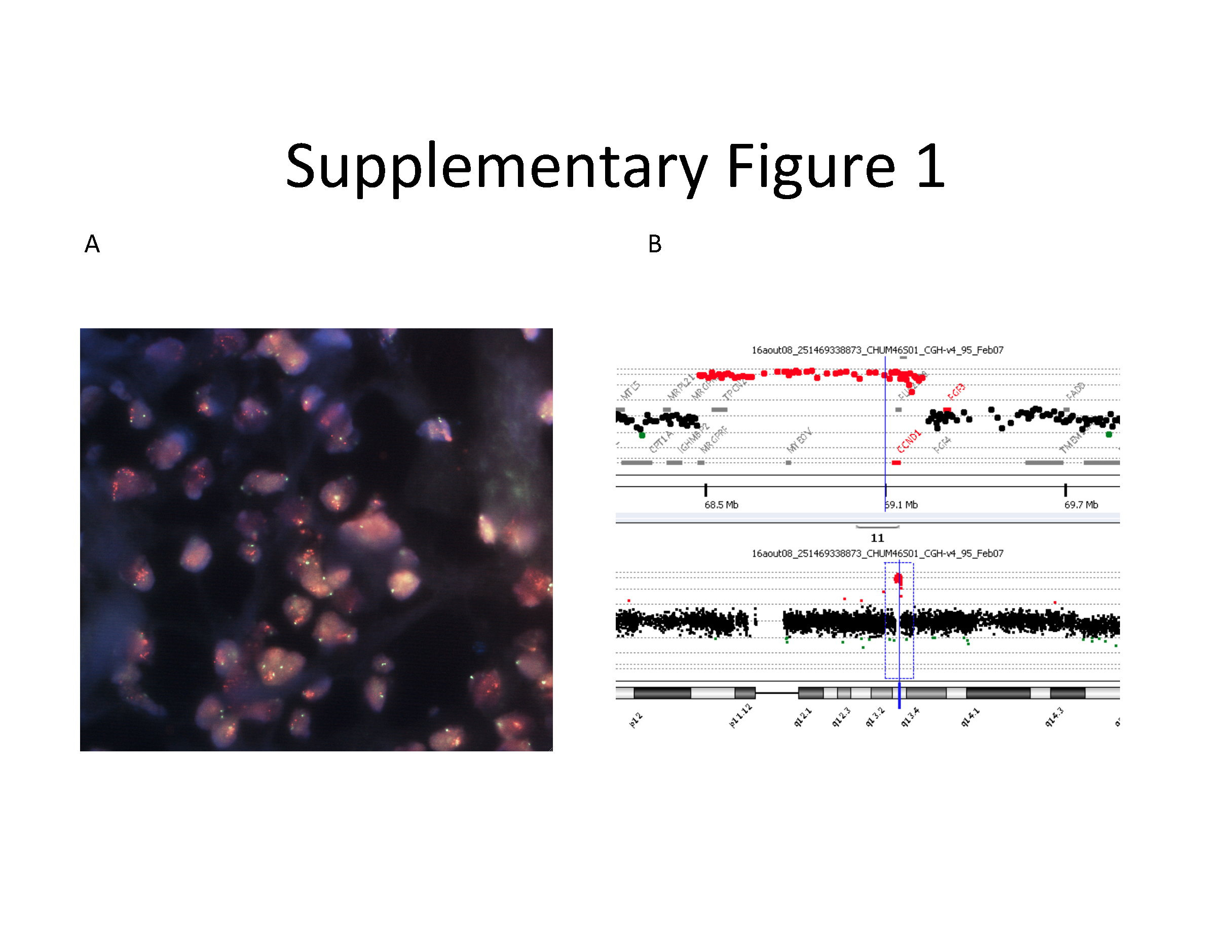

Supplement: Figure S1 — CCND1 amplification validation. Validation of a tumor with CCND1 gene amplification by FISH using array CGH data from DNA of the same tumor. FISH performed on a section from a paraffin-embedded formalin-fixed sample of a breast tumor (A) with red dots representing the CCND1 gene probe and green dots representing the centromeric 11q probe. (B) shows a chromosomal segment of chromosome 11 from a breast tumor that showed focal amplification of 11q13 including the CCND1 gene by array CGH (B). Red dots indicate probes with DNA copy number gain (values > log2=1). (TIF) [file pone.0081740.s001.tif]
