# Supplementary material for: RSF1 and Not Cyclin D1 Gene Amplification May Predict Lack of Benefit from Adjuvant Tamoxifen in High-Risk Pre-Menopausal Women in the MA.12 Randomized Clinical Trial
Source: PLoS One. 2013 Dec 19;8(12):e81740. doi: 10.1371/journal.pone.0081740 (PMC3868649; doi:10.1371/journal.pone.0081740)
Supplement: Table S1 — Patient characteristics by CCND1 status. Baseline characteristics for patients with non-amplified (“Low CCND1”) and amplified (“High CCND1”) CCND1. (PDF) [file pone.0081740.s002.pdf]

Table S1. Patient characteristics by CCND1 status.

Characteristic	Low CCND1		High CCND1	
	# of patients	% of patients	# of patients	% of patients
**Receptor status (p=0.14* for receptor status; p=0.01 for ER status; p=0.35 for PR status)**				
ER and/or PR positive	294	73	33	87
ER and PR negative	48	12	1	3
ER negative and PR unknown	59	15	4	10
ER positive	254	63	32	84
ER negative	147	37	6	16
PR positive	165	41	12	32
PR negative	63	16	5	13
PR unknown	173	43	21	55
**Nodal status (p=0.15)**				
Node-negative	105	26	4	10
1-3 nodes	217	54	27	71
4-9 nodes	69	17	6	16
10+ nodes	10	3	1	3
**Adjuvant chemotherapy (p=0.29)**				
CEF	98	24	5	13
CMF	175	44	19	50
AC	128	32	14	37
**Age (years) (p=0.64)**				
Median	45.7		45.0	
Range	29.3-57.8		27.1-54.1	
**Stage (pathologic) (p=0.39)**				
I	39	10	2	5

II	336	84	35	92
III	26	6	1	3
**Pathologic T stage (p=0.67)**				
1	167	42	15	39
2	204	51	22	58
3/4	30	7	1	3
**ECOG status (p=0.04)**				
0	268	67	23	61
1	130	32	13	34
2	3	1	2	5
**Treatment (p=0.49)**				
Tamoxifen	198	49	21	55
Placebo	203	51	17	45

* p-values refer to difference between high and low CCND1 groups by chi-square test except for age, for which a Wilcoxon two-sample test was used.
