# Supplementary material for: RSF1 and Not Cyclin D1 Gene Amplification May Predict Lack of Benefit from Adjuvant Tamoxifen in High-Risk Pre-Menopausal Women in the MA.12 Randomized Clinical Trial
Source: PLoS One. 2013 Dec 19;8(12):e81740. doi: 10.1371/journal.pone.0081740 (PMC3868649; doi:10.1371/journal.pone.0081740)
Supplement: Table S2 — Patient characteristics by RSF1 status. Baseline characteristics for patients with non-amplified (“Low RSF1”) and amplified (“High RSF1”) RSF1. (PDF) [file pone.0081740.s003.pdf]

Table S2. Patient characteristics by RSF1 status.

Characteristic	Low RSF1		High RSF1	
	# of patients	% of patients	# of patients	% of patients
**Receptor status (p=0.13* for receptor status; p=0.05 for ER status; p=0.75 for PR status)**				
ER and/or PR positive	277	73	25	89
ER and PR negative	46	12	2	7
ER negative and PR unknown	59	15	1	4
ER positive	243	64	23	82
ER negative	139	36	5	18
PR positive	144	38	10	36
PR negative	61	16	6	21
PR unknown	177	46	12	43
**Nodal status (p=0.31)**				
Node-negative	97	25	3	11
1-3 nodes	211	55	17	61
4-9 nodes	64	17	7	25
10+ nodes	10	3	1	3
**Adjuvant chemotherapy (p=0.80)**				
CEF	89	23	5	18
CMF	168	44	13	46
AC	125	33	10	36
**Age (years) (p=0.95)**				
Median	45.5		45.5	
Range	29.3-56.6		27.1-57.8	
**Stage (pathologic) (p=0.47)**				
I	36	10	1	3
II	322	84	26	94
III	24	6	1	3
**Pathologic T stage (p=0.10)**				
1	157	41	11	39
2	199	52	15	54

3/4	26	7	2	7
**ECOG status (p=0.08)**				
0	264	69	14	50
1	114	30	14	50
2	4	1	0	0
**Treatment (p=0.52)**				
Tamoxifen	188	49	12	43
Placebo	194	51	16	57

* p-values refer to difference between high and low RSF1 groups by chi-square test except for age, for which a Wilcoxon two-sample test was used.
